# Early detection of optic nerve head changes using optical coherence tomography after using mesenchymal stromal cells as intravitreal therapy in rabbit models of ocular hypertension

**DOI:** 10.14202/vetworld.2024.500-508

**Published:** 2024-02-29

**Authors:** Karine dos Santos Evangelho, Carlos Cifuentes-González, William Rojas-Carabali, Clemencia De Vivero-Arciniegas, Mariana Cañas-Arboleda, Gustavo Salguero, Carolina Ramírez-Santana, Alejandra de-la-Torre

**Affiliations:** 1 Doctoral Program in Biomedical and Biological Sciences, School of Medicine and Health Sciences, Universidad del Rosario, Bogotá, Colombia; 2 Neuroscience (NEUROS) Research Group, Neurovitae Research Center, Institute of Translational Medicine (IMT), School of Medicine and Health Sciences, Universidad del Rosario, Bogotá, Colombia; 3 Higher School of Ophthalmology- -Instituto Barraquer de América, Bogotá, Colombia; 4 Advanced Therapies Unit, Instituto Distrital de Ciencia Biotecnología e Innovación en Salud-IDCBIS, Bogotá, Colombia; 5 Center for Autoimmune Diseases Research (CREA), School of Medicine and Health Sciences, Universidad del Rosario, Bogotá, Colombia

**Keywords:** glaucoma, mesenchymal stromal cell, ocular hypertension, optic nerve head, optical coherence tomography, rabbit

## Abstract

**Background and Aim:**

Stem cell therapy is considered a promising treatment for several neurodegenerative diseases. However, there are very few studies on the use of this therapy in glaucoma models. By detecting the changes produced by glaucoma early, cell therapy could help prevent the events that lead to blindness. In this study, early changes in the optic nerve head (ONH) as detected by optical coherence tomography (OCT) after the application of human Wharton’s jelly-derived mesenchymal stromal cells (hWJ-MSCs) in an experimental model of ocular hypertension (OH) were evaluated.

**Materials and Methods:**

Fifteen New Zealand rabbits were randomly divided into the following three groups: G1: OH, G2: hWJ-MSCs, and G3: OH + hWJ-MSCs. An OH model was constructed, and the intraocular pressure (IOP) was measured regularly. At week 7, 10^5^/100 μL hWJ-MSCs were intravitreally injected. Retinography and OCT were used to evaluate structural changes in ONH.

**Results:**

IOP increased significantly in G1 and G3 from week 3 onward. Retinography revealed more significant optic nerve changes, that is, papillary asymmetry suggestive of optic nerve excavation, vascular alterations, and irregular hypopigmentation peripheral to the optic disk margin, in G1 compared with G3. OH locates the hWJ-MSCs solution in the vitreous in front of the optic nerve. OCT revealed retinal nerve fiber layer (RNFL) reduction in all groups, reduced optic cup volume in G2 and G3 between weeks 1 and 9, and significant ganglion cell layer thickness reduction in G1 and a slight increase in G3.

**Conclusion:**

Intravitreal hWJ-MSCs injection produced changes in optic cup volume, which were detected early on by OCT; however, RNFL could not be restored in this OH model.

## Introduction

Glaucoma is a neuropathy characterized by a loss of the retinal nerve fiber layer (RNFL) and alterations in the optic nerve head (ONH), resulting in visual loss [1]. Intraocular hypertension is the main modifiable risk factor for glaucoma; it can lead to ONH cupping and RNFL thinning when the disease is moderately advanced. Diagnosing the early stages of glaucoma can be challenging due to the misinterpretation of changes in ONH and RNFL during its initial stages, leading to clinical misdiagnosis [2]. Early detection and treatment of glaucoma are essential for preventing disease progression; neuroprotective therapies can be used to prevent rapid and irreversible cascade of events that ultimately lead to blindness [3]. Optical coherence tomography (OCT) can provide an objective and reliable structural assessment of ONH and RNFL because they are important structures for early diagnosis and disease monitoring. OCT images in previous studies have accurately reflected glaucoma histopathology in animal models of retinal disease [4, 5].

Although there is no cure for glaucoma, early treatment can often delay the damage and protect vision [6, 7]. Using stem cells as a neuroprotective agent for retinal ganglion cells (RGCs) provides a promising approach [8]. In this context, the use of neuroprotective therapies in glaucoma could be beneficial in preventing neuronal damage, complementing standard treatment, and enhancing patients’ quality of life by helping to preserve functional vision and reducing the risk of irreversible visual impairment. Mesenchymal stromal cells (MSCs) can be used as a neuroprotective therapy due to their multipotent self-replication ability, low immunogenicity, and ease of isolation and expansion [9]. Also, their paracrine properties [10] are known to secrete various neuroprotective proteins that promote damaged RGC survival and regeneration in eyes affected by glaucoma [11]. Numerous studies have reported that MSCs can mitigate retinal inflammation promoted by T-cells, thereby providing protective benefits for damaged retinal tissue [8]. However, the use of MSCs in an immunosuppressive environment, such as glucocorticoid (GC)-induced ocular hypertension (OH), has not yet been reported.

Therefore, the aim of this study was to use OCT and retinography to examine the initial effects on ONH produced by GCs following intravitreal application of MSCs in an OH model.

## Materials and Methods

### Ethical approval

All experimental and animal care procedures were conducted in accordance with the Association for Research in Vision and Ophthalmology Statement for the Use of Animals in Ophthalmic and Vision Research and approved by the Animal Ethics Committee of the Higher School of Ophthalmology - Instituto Barraquer de América – reference C-20190226-1. The Animal Research: Reporting of *in vivo* Experiments guidelines were followed in this study.

### Study period and location

This study was conducted from March to July 2021 at the Higher School of Ophthalmology - Instituto Barraquer de América, Colombia.

### Animals

The rabbits used in this study were provided by Sentagro E.A.T./NIT: 900131278-7. The study used 15 New Zealand male rabbits aged approximately 4 months and weighing 2–3 kg. Animals were housed according to National Institutes of Health guidelines. Before inclusion in this study, all animals underwent a complete ocular examination to rule out the presence of ocular disease. Intraocular pressure (IOP) measurements were performed by applanation tonometry (Tono-Pen AVIA Vet/Reichert, Depew, New York, USA), slit-lamp biomicroscopy of the anterior segment (SL-15/Kowa, Tokyo, Japan), direct ophthalmoscopy (Welch Allyn, Skaneateles Falls, WA, USA), and indirect ophthalmoscopy (Vantage Plus binocular, Keeler, UK).

Animals suffering from ocular surface diseases such as chronic keratitis or conjunctivitis of any etiology (including dry eye syndrome and systemic inflammatory disorders) and opacity of the lens were excluded from this study. Three intervention groups with five animals each were assigned as follows: Group 1 contained animals suffering OH, Group 2 rabbits receiving human Wharton’s jelly-derived mesenchymal stromal cells (hWJ-MSCs), and Group 3 rabbits receiving hWJ-MSCs (OH+hWJ-MSCs).

### Anesthesia procedure

The rabbits were anesthetized with an intramuscular injection of 35 mg/kg ketamine (Ketafine, Brouwer, Argentina) and 8 mg/kg xylazine (2% Xilacina, Erma, Colombia) before receiving an intravitreal injection of hWJ-MSCs. A 0.5% proparacaine anesthetic eye drop (Alcaine, Alcon, Barcelona) was used before subconjunctival GC injection. Artificial tears (Splash Tears, Sophia, Colombia) were instilled daily to reduce possible complications related to topical anesthesia. A slit lamp was used to analyze the ocular surface to detect possible corneal alterations (topical anesthesia was used).

### OH Induction

OH was induced in ten eyes, five eyes G1, and five eyes G3. Topical prednisolone acetate drops (10 mg/mL Prednefrin Forte Eye drops, Allergan, Brazil) were used twice a day to raise and maintain elevated IOP. This was associated with weekly subconjunctival injections of 0.5 mL betamethasone acetate (Celestone Chronodose, 3 + 3 mg/mL disodium phosphate, Schering-Plough, Mexico) [12, 13] in the right eye for 5 weeks, with the left eye used as the control.

### Measurement of IOP

An applanation tonometer (Tono-Pen AVIA Vet/Reichert) was used to measure both eyes’ IOP for 9 consecutive weeks. IOP was measured twice daily (at 7:00 am and 4:00 pm) in both eyes of all the experimental animals. The animals had minimal head and neck restraint; excessive pressure on the eyelids and neck was avoided. Five readings were taken for each eye and then averaged. The starting eye was randomly selected and tonometry was performed by the same examiner in all cases. OH was considered to be >15 mmHg, based on normal IOP in rabbits [14].

### Isolation and culturing hWJ-MSCs

Umbilical cord samples were collected from full-term births (cesarean and vaginal deliveries). Informed consent was obtained from healthy donors before samples were collected (acceptance reference 2019EE44993). Bogotá’s District Secretary of Health’s Ethics Committee approved the informed consent form. hWJ-MSCs were isolated as previously described by Silva-Cote *et al*. [15] and Cañas-Arboleda *et al*. [16]. Umbilical cord fragments were washed in 0.9% saline solution with 10,000 U/mL 1% penicillin/streptomycin, and umbilical veins, arteries, and outer membranes were removed. Wharton’s jelly was minced and cultured in flasks containing Dulbecco’s Modified Eagle’s Medium low glucose (Life Technologies, Carlsbad, CA, USA), 10,000 U/mL 1% penicillin/streptomycin supplemented with 10% human platelet lysate plus 8 IU/mL heparin. hWJ-MSCs cultures were maintained at 37°C in a humidified atmosphere containing 5% CO_2_. Cultures with 80% confluency were harvested, and hWJ-MSCs expanded until passage 3 was used in subsequent experiments.

### hWJ-MSCs quality control

hWJ-MSCs were immunophenotypically characterized by labeling the cells with anti-CD90-APC, -CD73-PE/Cy7, -CD105-PE, -CD274-PE, -CD45-APC/Cy7, -CD34-PerCP- Cy5.5, and -CD31-PE antibodies (Biolegend, San Diego, USA). T-cell depletion lymphocytes (TCD3^+^) inhibition was evaluated and cell suspensions were analyzed for sterility, endotoxins, and mycoplasma. A FACSCanto II cytometer (BD, Franklin Lakes, NJ, USA) and FlowJo vX.7.0 software (TreeStar, USA) was used for flow cytometry and data analysis, respectively.

### Intravitreal injection of hWJ-MSCs

Animals in groups 2 and 3 received a single intravitreal injection of hWJ-MSCs suspension at week 7 of the study. hWJ-MSCs were supplied in single-dose cryotube vials that had been previously thawed. After general anesthesia in sterile conditions, the hWJ-MSCs suspension (1 × 10^5^ cells/100 μL) [17] was gently injected into the vitreous cavity with a 30-gauge needle entering the globe through the pars plana in the direction of the optic disk. A sterile irrigating solution (balanced salt solution) was then intravitreally injected into the control eye (left).

### Retinography

A Zeiss Ff-450 Plus IRu (FF 450*^plus^* Fundus Camera with VISUPAC™ Digital Imaging System, Zeiss, German, UK) fundus camera was used to take photographs of the right eye fundus at weeks 1 and 9 of all the animals studied. The rabbits were carefully held to capture the images and their eyes were placed close to 10 cm from the photographic equipment. Anatomical parameters, that is, papillary asymmetry (observation of optic cup diameter and optic disk diameter), were used as indicators of optic nerve abnormalities between weeks 1 and 9 of the study. The animals were not anesthetized for this study.

### OCT images

All examinations were performed in dilated eyes (1% tropicamide (Mydriacyl, Alcon, Colombia)), and the corneas were kept moist with artificial tears (Splash Tears). A Zeiss Cirrus HD-OCT (Software version 3.0.0.64; Carl Zeiss Meditec, Dublin, CA, USA) was used for imaging based on protocols described for the species [5, 18, 19]. Photographs were obtained from conscious animals using minimal manual restraint; all animals’ retinal and optic nerve images were recorded. Paracentral B-scans located 3 mm ventral optic disk (visual streaks) to the ONH were acquired. Only scans with an intensity signal >5 and without eye movements were used for analysis; 5 µm axial and 15 µm transversal scans were performed. Scan circles and caliper functions were fitted for ONH measurements. We analyzed the following ONH parameters: average RNFL thickness, disk area, and the vertical and horizontal relationship between the cup, disk, and cup volume. The ganglion cell layer (GCL) was measured in visual streaks using a macular cube; OCT variations were compared throughout the study period.

### Statistical analysis

Data analysis was performed using a Jamovi statistical spreadsheet (version 2) (Jamovi, 2021; https://www.jamovi.org/); the results are presented as means, standard deviations, and interquartile ranges. The Shapiro-Wilk normality test was used to determine the changes in data distribution by group during weekends. Repeated-measures analysis of variance (ANOVA) was used in cases of normal distribution, whereas Friedman test was used for non-normal distributions. Repeated measure ANOVA detected significant differences. Tukey and Bonferroni *post hoc* tests were used for further analyses. Thus, it was not necessary to check for the assumption of homoscedasticity since this assumption was consistently met. Depending on the distribution, a paired sample t-test or paired sample Wilcoxon test was used for analysis involving incomplete follow-up with OCT.

When statistically significant differences were identified by Friedman test, the Durbin–Conover test was used to compare groups. One-way ANOVA or Kruskal–Wallis one-way ANOVA was used to analyze the differences between the groups over the weeks, depending on distribution. Levene’s test for homoscedasticity was used if one-way ANOVA revealed statistically significant differences; a *post hoc* analysis test was used based on its results (i.e., Tukey and Games-Howell for equal variances and different variances). The Dwass–Steel–Critchlow–Fligner test was used to explore the differences between groups revealed by Kruskal–Wallis ANOVA. Pearson’s or Spearman’s correlation tests regarding IOP and RNFL were used based on data distribution.

## Results

### hWJ-MSCs quality control

hWJ-MSCs induced the expression (80%) of antibodies such as cluster of differentiation (CD90, CD73, CD105, and CD274, but not CD45, CD34, CD31), and/or human leukocyte antigen - DR isotype (HLA-DR) expression (≥2%); the TCD3+ inhibition capacity was ≤87%. The cells had 99% viability and endotoxin levels of <0.45 EU/mL and lacked mycoplasma and other microorganisms (sterile cellular suspension).

### IOP in an early glaucoma model

IOP significantly increased (p < 0.001, Kruskal–Wallis ANOVA) in G1 and G3 from week 3 of the study (12.42 ± 1.85 [7.0–21.0] mmHg) compared with that in the control group (11.59 ± 1.89 [7.0–15.0] mmHg). There were no significant differences in control eyes between weeks (p > 0.05, Kruskal–Wallis ANOVA). Right eye IOP was 25.22 ± 12.31 (13.0–64.0) mmHg, 13.33 ± 3.32 (7.0–25.0) mmHg, and 19.16 ± 4.47 (11.0–42.0) mmHg in G1, G2, and G3, respectively. After discontinuing GC treatment at week 5, a sudden increase in G1 IOP was observed compared to that for G3 (Supplementary Table-1). All groups had a slightly higher IOP in the afternoon than in the morning. However, this was not statistically significant, indicating that the animals were suitably acclimatized (p > 0.05, Kruskal–Wallis ANOVA).

**Supplementary Table-1:** Measurements of the IOP in different groups on weeks.

IOP	Week 1 Media (SD) (IQR)	Week 2 Media (SD) (IQR)	Week 3 Media (SD) (IQR)	Week 4 Media (SD) (IQR)	Week 5 Media (SD) (IQR)	Week 6 Media (SD) (IQR)	Week 7 Media (SD) (IQR)	Week 8 Media (SD) (IQR)	Week 9 Media (SD) (IQR)
G1	12.8 (2.0) (7.0–19.0)	10.5 (2.5) (8.0–17.0)	14.3 (2.2) (9.0–20.0)	15.1 (2.2) (8.0–21.0)	15.5 (3.3) (8.0–26.0)	22.0 (7.8) (13.0–66.0)	25.2 (12.3) (13.0–64.0)	23.8 (14.4) (11.0–74.0)	18.1 (9.9) (12.0–45.0)
G2	12.3 (1.9) (7.0–17.0)	11.1 (2.4) (7.0–16.0)	10.8 (1.6) (7.0–16.0)	12.0 (1.8) (9.0–17.0)	12.9 (1.6) (9.0–15.0)	12.3 (3.3) (7.0–15.0)	12.7 (2.2) (8.0 15.0)	12.2 (1.1) (8.0–15.0)	11.6 (1.5) (9.0–15.0)
G3	12.2 (2.1) (7.0–19.0)	10.2 (3.1) (8.0–17.0)	14.2 (3.3) (9.0–24.0)	15.3 (3.9) (8.0–30.0)	15.2 (4.0) (8.0–33.0)	18.2 (3.9) (10.0–29.0)	19.2 (4.5) (11.0–42.0)	17.2 (4.3) (7.0–28.0)	13.2 (3.2) (7.0–25.0)

ANOVA one way; *Kruskal-Wallis ANOVA. G1: ocular hypertension, G2: hWJ-MSCs; G3: ocular hypertension + hWJ-MSCs; SD: standard deviation; IQR: interquartile range. IOP: intraocular pressure.

### Ophthalmological and retinographic changes and hWJ-MSCs use post-hypertension

A complete ophthalmological examination was performed every day before OH was induced to identify any ophthalmological changes occurring during the development of the proposed experimental model. No alterations in the cornea’s curvature or irregularities in the corneal surface were observed with topical anesthesia when the anterior segment was evaluated using a slit-lamp.

No damage to the eyeball adnexal organs or blepharospasm, epiphora, or photophobia, which could indicate pain or discomfort, was observed during the 5 weeks of OH induction in groups G1 and G3. However, one G3 animal suffered intense conjunctival hyperemia, mild corneal edema, rubeosis iridis, and iritis from week 7 of the study after applying hWJ-MSCs. Clinical characteristics changed by week 9 (less conjunctival hyperemia, gray-green iris, and corneal edema). At week 9, one animal in G2 had mydriasis without response to light stimulation. No signs of uveitis or infection were observed after intravitreal application of hWJ-MSCs in the other experimental animals.

**Figure-1:**
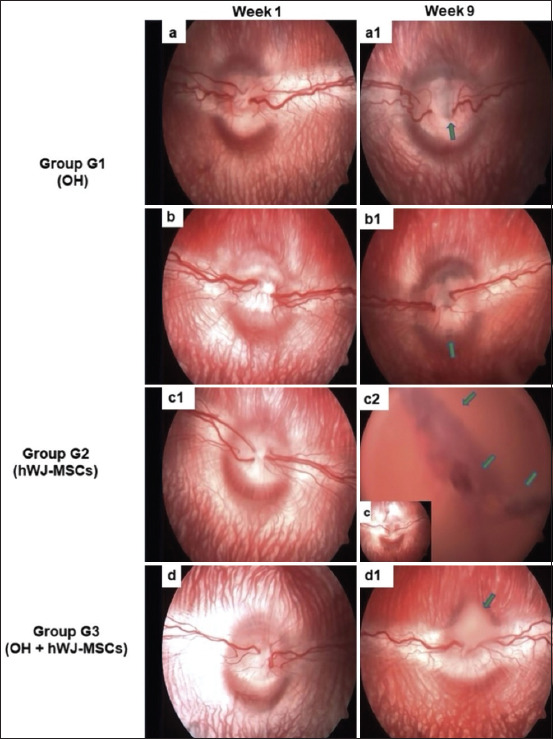
Fundus photographs taken 1 and 9 weeks after inducing ocular hypertension (OH) and intravitreal hWJ-MSCs transplant. The results observed for G1 revealed more significant changes in the optic nerve (a1: Papillary asymmetry suggestive of optic nerve excavation; b1: Irregular hypopigmentation located peripheral to the optic disc margin) compared to G2 (c1: Other animals exhibited no alterations; c2: Vitreous hemorrhage in one animal) and G3 (d1: hWJ-MSCs solution localized on the optic nerve head). a, b, c, d: Eyes week 1. G1 OH, G2 hWJ-MSCs and G3 OH + hWJ-MSCs. hWJ-MSCs=Human Wharton’s jelly-derived mesenchymal stromal cells.

Retinography revealed vascular dilatation, tortuosity, and ONH changes, including G1 papillary asymmetry. Vitreous hemorrhage was observed in the same animal in G2, which suffered from mydriasis; no changes were observed in the other animals. No retinal or optic disk abnormalities in the posterior segment were observed in G3 rats compared with those in the G1 rats; however, an MSCs-enriched solution located over the optic nerve was observed in one animal in this group (Figure-1).

### Optic nerve OCT

Structural changes in retinal and ONH were observed in weeks 1 and 9 after OH and intravitreal injection of hWJ-MSCs. A decrease in RNFL thickness was observed in G1 at week 9 (40.5 ± 20.3 [25.0–69.0] µm) compared to week 1 (49.4 ± 17.6 [29.0–68.0] µm) and week 4 (58.2 ± 9.7 [48.0–69.0] µm) (p = 0.159, Friedman’s ANOVA). A decrease in RNFL thickness in G3 was also observed at week 9 (31.4 ± 14.4 [11.0–51.0] µm) compared to weeks 1 (54.2 ± 25.5 [29.0–83.0] µm) and 4 (36.0 ± 12.1 [19.0–53.0] µm) (p = 0.249, Friedman’s ANOVA). A decrease in RNFL thickness was observed in G2 when comparing week 1 (60.8 ± 24.2 [32.0–95.0] µm) and week 9 (43.0 ± 18.3 [21.0–60.0] µm, p = 0.393, Wilcoxon test); such a decrease was not significant.

A decrease in G1 was observed by measuring the disk area at week 9 (2.0 ± 0.4 [1.5–2.4] mm^2^) compared to weeks 1 (2.9 ± 1.5 [1.4–4.7] mm^2^) and 4 (2.7 ± 0.8 [2.2–4.2] mm^2^) (p = 0.247, Friedman’s ANOVA). A decrease in G3 disk area was also observed at week 9 (3.1 ± 0.4 [2.5–3.6] mm^2^) compared with weeks 1 (3.3 ± 0.6 [2.8–4.2] mm^2^) and 4 (3.3 ± 0.7 [2.1–3.7] mm^2^) (p = 0.802, Friedman’s ANOVA). However, an increase in disk area was observed in G2 when comparing weeks 1 (2.8 ± 1.1 [1.8–4.0] mm^2^) and 9 (3.0 ± 1.0 [2.0–4.3] mm^2^), p = 0.555, Wilcoxon test.

**Figure-2:**
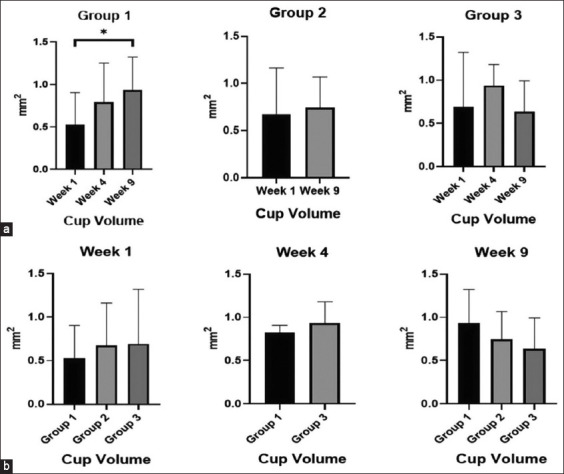
Changes in optic nerve after inducing ocular hypertension (OH) and intravitreal hWJ-MSCs transplant. (a) Analysis of optic cup volume in the groups during the weeks of the study and (b) Comparing the groups’ optic cup volume. Repeated measures analysis of variance (ANOVA); *Friedman’s ANOVA. p < 0.05. G1 OH, G2 hWJ-MSCs and G3 OH + hWJ-MSCs. hWJ-MSCs=Human Wharton’s jelly-derived mesenchymal stromal cells.

A significant decrease in GCL thickness was observed in G1 at week 9 (8.5 ± 5.2 [3.0–15.0] µm) compared with that at weeks 1 (35.4 ± 14.94 [20.0–58.0] µm) and 4 (21.8 ± 6.1 [16.0–32.0] µm) (p = 0.044, Friedman’s ANOVA), but it was not statistically significant in G2 compared with that at weeks 1 (25.4 ± 9.9 [16.0–38.0] µm) and 9 (16.5 ± 12.6 [4.0–32.0] µm p = 0.1, Wilcoxon test. However, an increase in GCL thickness in G3 was observed at week 9 (20.4 ± 9.2 [8.0–31.0] µm) compared to weeks 1 (17.4 ± 12.12 [6.0–37.0] µm) and 4 (16.0 ± 19.4 [2.050.0] µm) (p = 0.249, Friedman’s ANOVA). No statistically significant differences in GCL thickness between the groups were observed in the *post* *hoc* test. Regarding optic cup volume analysis, a significant increase in G1 was observed when comparing weeks 1 and 9 (p = 0.047, Tukey) (Figures-2a and b). This difference was not statistically significant regarding changes in G1 regarding optic cup volume in the *post* *hoc* analysis (p = 0.052, Scheffe and p = 0.07, Bonferroni). However, reduced optic cup volume was observed between weeks 1 and 9 in G2 (p = 0.502) and G3 (p = 0.445). There was no statistically significant correlation between RNFL and IOP.

Differences regarding RNFL were observed between the G1 and G3 groups and the control group at week 4 when comparing the study groups (Tables-1-3) (p = 0.043, Kruskal–Wallis ANOVA.). Differences were observed between G1 and G3 in *post* *hoc* analysis (p = 0.049, Tukey) (Table-2). Statistically significant differences were found between groups in week 9 regarding disk area (p = 0.024); statistically significant differences between groups were lost in *post hoc* analysis by Tukey test (Table-3) (p = 0.043, Kruskal–Wallis ANOVA; Tables-1-3). *Post hoc* analysis revealed differences between G1 and G3 (p = 0.049, Tukey) (Table-2). Statistically significant differences in disk area were found between the groups at week 9 (p = 0.024); these differences were lost in *post hoc* analysis by Tukey’s test (Table-3).

**Table-1:** Measurements of the optic nerve head in different groups in week one, performed by Cirrus HD-OCT.

Optic Nerve OCT	G1 Media (SD) (IQR)	G2 Media (SD) (IQR)	G3 Media (SD) (IQR)	p-value
RNFL (µm)	49.4 (17.6) (29.0–68 0.0)	60.8 (24.2) (32.0–95.0)	54.2 (25.5) (29.0–83.0)	0.725
Disk Area (mm^2^)	2.9 (1.5) (1.4–4.7)	2.8 (1.1) (1.8–4.0)	3.3 (0.6) (2.8–4.2)	0.622
Coup Volume	0.5 (0.4) (0.2–1.0)	0.7 (0.5) (0.2–1.2)	0.7 (0.6) (0.0–1.5)	0.831
Vertical Coup/Disc Ratio	0.8 (0.1) (0.6–0.9)	0.9 (0.1) (0.8–1.0)	0.8 (0.3) (0.3–1.0)	0.277*
GCL (µm)	35.4 (14.9) (20.0–58.0)	25.4 (9.9) (16.0–38.0)	17.4 (12.1) (6.0–37.0)	0.196

ANOVA one way;

* Kruskal-Wallis ANOVA. G1: ocular hypertension, G2: hWJ-MSCs; G3: ocular hypertension + hWJ-MSCs; SD: standard deviation; IQR: interquartile range. RNFL: Retinal Nerve Fiber Layer; GCL: Ganglion Cell Layer

**Table-2:** Measurements of the optic nerve head in different groups in week four, performed by Cirrus HD-OCT.

Optic Nerve OCT	G1 Media (SD) (IQR)	G3 Media (SD) (IQR)	Control Media (SD) (IQR)	p-value
RNFL (µm)	58.2 (9.7) (48.0–69.0)	36.0 (12.1) (19.0–53.0)	45.0 (16.6) (33.0–72.0)	0.043
Disk Area (mm^2^)	2.7 (0.8) (2.2–4.2)	3.3 (0.7) (2.1–3.7)	3.7 (0.7) (2.7–4.2)	0.136*
Coup Volume	0.8 (0.5) (0.3–1.4)	0.9 (0.2) (0.5–1.1)	0.9 (0.5) (0.2–1.4)	0.826
Vertical Coup/Disc Ratio	0.8 (0.1) (0.7–0.9)	0.9 (0.0) (0.8–0.9)	0.8 (0.0) (0.8–0.9)	0.234
GCL (µm)	21.8 (6.1) (16.0–32.0)	16.0 (19.4) (2.0–50.0)	29.8 (16.5) (10.0–47.0)	0.274*

ANOVA one way;

* Kruskal-Wallis ANOVA. G1: ocular hypertension, G3: ocular hypertension + hWJ-MSCs; Controls are mixed of the three groups. SD: standard deviation; IQR: interquartile range. RNFL: Retinal Nerve Fiber Layer; GCL: Ganglion Cell Layer

**Table-3:** Measurements of the optic nerve head in different groups in week nine, performed by Cirrus HD-OCT.

Optic Nerve OCT	G1 Media (SD) (IQR)	G2 Media (SD) (IQR)	G3 Media (SD) (IQR)	p-value
RNFL (μm)	40.5 (20.3) (25.0–69.0)	43.0 (18.3) (21.0–60.0)	31.4 (14.4) (11.0–51.0)	0.589
Disk Area (mm^2^)	2.0 (0.4) (1.5–2.4)	3.0 (1.0) (2.0–4.3)	3.1 (0.4) (2.5–3.6)	0.024
Coup Volume	0.9 (0.4) (0.6–1.4)	0.7 (0.3) (0.3–1.1)	0.6 (0.4) (0.1–1.1)	0.553
Vertical Coup/Disc Ratio	0.9 (0.1) (0.8–1.0)	0.8 (0.1) (0.7–1.0)	0.8 (0.2) (0.4–0.9)	0.704
GCL (μm)	8.5 (5.2) (3.0–15.0)	16.5 (12.6) (4.0–32.0)	20.4 (9.2) (8.0–31.0)	0.133

ANOVA one way; *Kruskal-Wallis ANOVA. G1: ocular hypertension, G2: hWJ-MSCs; G3: ocular hypertension + hWJ-MSCs; SD: standard deviation; IQR: interquartile range. RNFL: Retinal Nerve Fiber Layer; GCL: Ganglion Cell Layer

## Discussion

This study has shown that intravitreal hWJ-MSCs transplantation can lead to early detection of changes in ONH by OCT in an experimental model of OH. Structural improvement in optic cup volume was observed in this experimental model. In addition to secreting exosomes, which provide regenerative and restorative effects on the microenvironment [20, 21], MSCs secrete growth factors and cytokines that affect nearby cells.

Contrary to what He *et al*. [22] observed in a monkey model of OH, in a rabbit-based experimental model, the data presented here demonstrated that a detectable change in RNFL preceded the change produced in ONH. Furthermore, intravitreal injection of hWJ-MSCs did not prevent the progression of OH changes in RNFL.

Because glaucoma is an irreversible, progressive optic neuropathy, thinning of the RNFL is expected [23]. Therefore, a decrease in RNFL thickness was observed in all study groups when measuring RNFL by OCT, indicating that the intravitreal application of hWJ-MSCs did not affect the RNFL in the OH group. An incidental observation indicated a trend toward a reduction in RNFL thickness in the group that received cell transplants without OH. Although there was a trend toward reducing the thickness, it was not statistically significant. This observation regarding RNFL in this group may have been due to the release of cytokines and inflammatory mediators initially secreted by microglia cells [24] and should, therefore, not be considered innocuous.

Groups receiving cell therapy had decreased optic cup volume and increased optic nerve rim area was detected early by OCT. Such findings may have been associated with the increase in blood flow produced by cell therapy, thereby causing prelaminar tissue thickening and anterior movement of the lamina cribosa, thereby altering some ONH structures [25, 26]. MSCs have a direct effect on angiogenesis by increasing vascular endothelial growth factor and its receptor levels, suggesting that MSCs administration may provide a restorative microenvironment and exert a paracrine neuroprotective effect on RGCs [27].

Other acceptable explanations for such observations include prelaminar glial proliferation and connective tissue synthesis in response to intravitreally applied cell therapy. Although the optic cup volume and IOP decreased in the OH cell therapy group, no significant increase in RNFL thickness was observed. Hence, the explanation for this finding is probably not an increase in the number of axons, but rather a relaxation of the tissues supporting the papillary structures [28, 29] following cell therapy.

A trend toward greater RGC layer thickness and optic disk area maintenance was observed in the OH group transplanted with hWJ-MSCs compared to the group that did not receive these cells. This tendency to delay RGC loss has been described in the relevant literature regarding a rat optic nerve crush model involving intravitreal MSCs transplant [30, 31]. The secretion of anti-inflammatory molecules and trophic factors is one of the *in vivo* characteristics of hWJ-MSCs, which may explain the induced neuroprotection [32].

Degenerated axon regeneration in glaucoma is unlikely to occur in the remodeled cribriform plate [33, 34]. We believe that intravitreal hWJ-MSCs ameliorated the altered environment and promoted cellular neuroprotection in this study. Yang *et al*. [34] suggested that the mechanism underlying the axon regeneration-promoting effect after MSCs transplantation is likely due to cytokine or growth factor release, rather than differentiation into neural or glial cells.

Despite the significant differences found between the OH groups regarding IOP measurements, no significant alterations were observed regarding the various ONH measurements in OCT. Such observation means that IOP reduction was not the only factor conditioning ONH changes; variability concerning ONH changes to IOP reduction could have been due to the small sample size, the technique used or other coexisting modifying factors [35], that is, the use of cell therapy. In addition to the small sample size, which can reduce statistical power, the RGC layer measurement was a concern because the equipment used automatically takes the measurements, thereby not allowing image segmentation; this could have introduced subjective bias. Although OCT provided information regarding early optic nerve changes following cell therapy, histological analysis could be used to correlate *in vivo* OCT scan results with histopathological findings.

This study could help detect early histopathological characteristics of ONH alterations and provide greater security regarding hWJ-MSCs use. Future research should be aimed at correlating our optic nerve findings with RGC assessment using retinal whole mount plans stained with live RGC-specific staining and counting RGCs.

## Conclusion

Our study has shown that intravitreally applied cell therapy can produce changes in the ONH in an experimental OH model, aimed at early detection by OCT; however, such cell therapy failed to recover the RNFL in the short term. Therefore, further research into the role of hWJ-MSCs in the cellular microenvironment after transplantation is necessary to increase the understanding of glaucoma pathogenesis and develop new and better treatment approaches.

## Authors’ Contributions

KSE and AT: Research concept and design and drafted the manuscript. KSE: Collected and assembled data. KSE, CCG, and WRC: Data analysis and interpretation. KSE, AT, CCG, and WRC: Critical revision of the manuscript. KSE, AT, CDVA, GS, MCA, and CRS: Methodology. All authors compiled, read, revised, and approved the final manuscript.
